# Human motor decoding from neural signals: a review

**DOI:** 10.1186/s42490-019-0022-z

**Published:** 2019-09-03

**Authors:** Wing-kin Tam, Tong Wu, Qi Zhao, Edward Keefer, Zhi Yang

**Affiliations:** 1 Department of Biomedical Engineering, University of Minnesota Twin Cities, 7-105 Hasselmo Hall, 312 Church St. SE, Minnesota, 55455 USA; 2 Department of Computer Science and Engineering, University of Minnesota Twin Cities, 4-192 Keller Hall, 200 Union Street SE, Minnesota, 55455 USA; 3 Nerves Incorporated, Dallas, TX P. O. Box 141295 USA

**Keywords:** Motor decoding, Brain-machine interfaces, Neuroprosthesis, Neural signal processing

## Abstract

Many people suffer from movement disability due to amputation or neurological diseases. Fortunately, with modern neurotechnology now it is possible to intercept motor control signals at various points along the neural transduction pathway and use that to drive external devices for communication or control. Here we will review the latest developments in human motor decoding. We reviewed the various strategies to decode motor intention from human and their respective advantages and challenges. Neural control signals can be intercepted at various points in the neural signal transduction pathway, including the brain (electroencephalography, electrocorticography, intracortical recordings), the nerves (peripheral nerve recordings) and the muscles (electromyography). We systematically discussed the sites of signal acquisition, available neural features, signal processing techniques and decoding algorithms in each of these potential interception points. Examples of applications and the current state-of-the-art performance were also reviewed. Although great strides have been made in human motor decoding, we are still far away from achieving naturalistic and dexterous control like our native limbs. Concerted efforts from material scientists, electrical engineers, and healthcare professionals are needed to further advance the field and make the technology widely available in clinical use.

## Background

Every year, it is estimated that more than 180,000 people undergo some form of limb amputation in the United States alone [1]. In 1996, a national survey revealed that there are 1.2 million people living with limb loss [2]. The figure is expected to be more than tripled to 3.6 million by year 2050 [1]. Besides amputations, various neurological disorders or injuries will also affect one’s movement ability. Examples include spinal cord injury, stroke, amyotrophic lateral sclerosis, etc. Patients suffering from these conditions lose volitional movement control even though their limbs are still intact. No matter if it is amputation or neurological disorder, affected patients have their everyday life and work significantly disrupted. Some may be forced to give up their original jobs, while some may even lose the ability to take care of themselves entirely.

Fortunately, although part of the signal transduction pathway from higher cortical centers to muscles have been severed in those aforementioned conditions, in most of the cases we can still exploit the remaining parts to capture the movement intention of the subject. For amputation, the neurological pathway above the nerve stump is mostly intact. For neurological disorders and injuries, depending on the site of the lesion, usually upper stream structures are still intact and functioning. With modern neural interfacing technology, signal processing and machine learning algorithms, it is now possible to decode those motor intentions and use it to either replace the lost function (e.g. through a prosthesis) or to help rehabilitation (e.g. in stroke [3, 4]).

The signal for movement control can be intercepted at various points along the neural transduction pathway. Each of these points exhibits different features and poses unique advantages and challenges. Some of the methods are more invasive (e.g. intracortical recording) but also more versatile because they intercept neural signals at the upmost stream, so they are less reliant on the presence of residue functions. However, some others (e.g. surface electromyogram) while are less invasive, rely heavily on the presence of downstream functional structures and thus any upstream damages undermine their performance. Ultimately, the choice of signal modality to decode from depends on the location, type, and severity of the lesion. In this review, we will discuss the various opportunities available to decode motor intention from human subject at different locations along the motor control pathway. It is our hope that this comprehensive information can help make the most effective clinical decision on how to help the patients.

In this review, we will mainly focus on the decoding of motor intention on human subjects. Although animal studies are an very important and indispensable part of motor decoding research, the application on human subjects is the ultimate goal. Clinical trials on patients may introduce additional and non-negligible challenges to the system and experimental design. For example, in amputees or paralyzed subjects the ground-truth for limb movement is usually unavailable. Special considerations must be incorporated into the experimental design to work around this limitation. Furthermore, although some methods may be working very well on animal studies, their translation into human use may not be straightforward due to safety concerns or surgical difficulties. Therefore, a focus on human studies will allow us to have a more realistic expectation of the current state-of-the-art performance in the field. This knowledge can then in-turn better inform the decision choosing between risk and benefit of a decoding strategy.

## Main text

### Neurophysiology of motor control

To decode the motor intention of human subject, it is useful to first understand the natural neurophysiology of motor control, so that we may know where to intercept the control signal and what kind of signal feature that we may encounter.

Motor controls in the human body begins at the frontal and posterior parietal cortex (PPC) [5, 6]. These areas carry out high-level, abstract thinking to determine what actions to take in a given situation [7]. For example, when confronted with a player from the opposing team, a soccer player may need to decide whether to dribble, shoot or pass the ball to his teammate. The choice of the best action depends on the location of the player, the opponent and the ball. It also depends on the current joint angles of the knees and ankles in relation to the ball. The PPC receives input from the somatosensory cortex to get information on the current state of the body. It also has extensive interconnection with the prefrontal cortex, which is responsible for abstract strategic thoughts. The prefrontal cortex may need to consider other factors beside the sensory information about the current environment. For example, how skillful is the opponent compared to myself? What is the existing team strategy at the current state of the game, should I play more aggressively or defensively? The combination of sensory information, past experience, and strategic decision in the frontal and posterior parietal cortex determine what sequences of action to take.

The planning of the action sequence is then carried out by the premotor area (PMA) and the supplementary motor area (SMA), both located in Brodmann area 6 of the cortex. Stimulation in area 6 is known to elicit complex action sequence and intracortical recording in the PMA shows that it is activated around 1 second before movement and stops shortly after the movement is initiated [8]. Some neurons in the PMA also appear to be tuned to the direction of movement, with some of them only be activated when the hand move in one direction but not in the other.

After a sequence of action is planned in PMA or SMA, it requires input from the basal ganglia to actually initiate the movement. The basal ganglia contains the direct and indirect pathway [9–11]. The direct pathway helps select a particular action to initiate, while the indirect pathway filters out other inappropriate motor programs. In the direct pathway, the striatum (putamen and caudate) receives input from the cerebral cortex and inhibits the internal globus pallidus (GPi). In the resting state, GPi is spontaneously activated and inhibits the oral part of the ventral lateral nucleus (VLo) of the thalamus. Thus, inhibition of GPi will enhance the activity of VLo, which in turn excites the SMA. In the indirect pathway, the striatum excites GPi through the subthalamus nucleus (STN), which then suppresses VLo activity and in turn inhibits SMA. In some neurological disorder like Parkinson’s disease, deficit in the ability to activate the direct pathway will lead to difficulty in initiating a movement (i.e. bradykinesia), while deficit in the indirect pathway will lead to uncontrolled movement in the resting state (i.e. resting tremor).

After the basal ganglia helps filter out unwanted motor programs and focus on the selected programs, the primary motor cortex (M1) will be responsible for their low-level executions [12]. In the layer V of M1, there are population of large neurons pyramidal in shape that project their axon connections down the spinal cord through the corticospinal track. These axons connect with motor neurons in the spinal cord monosynaptically to activate muscles fibers. They also connect with inhibitory interneurons in the spinal cord to inhibit antagonistic muscles. This structure allows one single pyramidal cell to generate coordinated movement in multiple muscle groups.

Motor neurons in the spinal cord receive inputs from the M1 pyramidal cells through the corticospinal track [13]. They also receive the input indirectly from the motor cortex and cerebellum through the rubrospinal track, routed via the red nucleus in the midbrain. Although its functions is well established in lower mammal, the functions of the rubrospinal track in human appears to be rudimentary. Motor neurons in the ventral horn of the spinal cord bundle together to form the ventral root, which exits the spinal cord and joints with the dorsal root to form a mixed spinal nerve. The spinal nerve further branches out to smaller nerve fibers that innervate various muscles of the body. One motor neuron may supply multiple muscle fibers, collectively known as one motor unit. A muscle consists of multiple muscle fibers, grouped into motor units of various sizes, each of which may be supplied by different motor neurons. In large muscles such as those in the leg, one motor neuron may supply hundreds of muscle fibers. In smaller muscles, such as those in the fingers, one motor neuron may only supply 2 or 3 muscle fibers, enabling fine movement control.

**Fig. 1:**
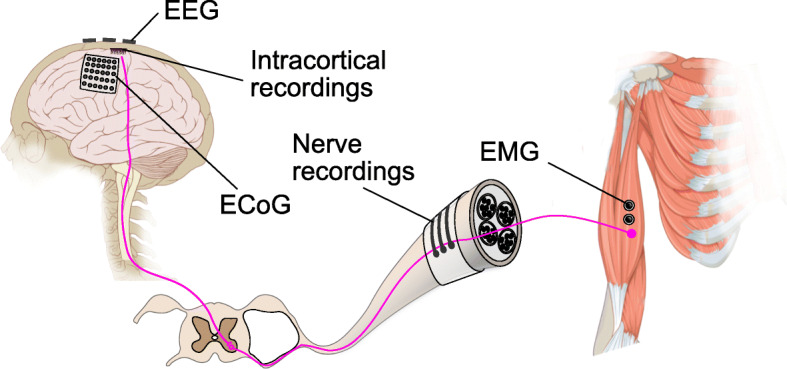
Overview of various ways to intercept motor control signals. Motor control signal is relayed from the primary motor cortex of the brain, via the spinal cord and peripheral nerve, to the muscle fibers. The control signal can be intercepted at various points using different techniques. Electroencephalography (EEG) captures the superimposed electrical fields generated by neural activity on the surface of of the scalp. Electrocorticography (ECoG) measures activity underneath the scalp on the surface of the brain. Intracortical recordings penetrate into the brain tissue to acquire multi- and single-unit activities. Electrodes can also be placed on the peripheral nerve to monitor the low level signal used to drive muscle contraction. Finally, electromyograph (EMG) can also be used to monitor the activity of the muscle directly (the figure contains elements of images adapted from Patrick J. Lynch and Carl Fredrik under Creative Commons Attribution License)

The motor control pathway of the human body goes from the high level associative area of the brain, mediated by the motor cortex, through the spinal cord to the individual muscle fibers. Each of the stages plays a different role and uses different mechanisms to ensure that a movement is carried out in a coordinated and smooth manner. Each of these stages also offers different signal modalities and features that can be exploited for motor decoding. We will now discuss these features and strategies to utilize them in details below. An overview showing the motor control pathway and various ways to intercept the control signal is shown in (Fig. 1).

### Cortical decoding of limb movements

All volitional motor controls originate from the brain. The motor cortex of the brain plays an especially important role in planning and executing motor commands. For some patients, the brain is the only site where motor intention can be captured because they have lost motor functions in all their extremities (e.g. in tetraplegic patients). Therefore, many efforts have been invested in cortical decoding.

#### Electroencephalography (EEG)

EEG is the measurement of weak electrical signals from the brain on the surface of the scalp. Its origin is believed to be the summation of postsynaptic potentials of excitable neural tissues in the brain [14]. The skull, dura and cerebrospinal fluid between the brain and the EEG electrodes attenuate the electrical signal significantly, thus the EEG signal is very weak, typically under 150 *μ*V. Those structures also act like temporal low-pass filters, limiting the useful bandwidth of the EEG signal to be below 100 Hz [15]. Furthermore, due to the volume conduction effect of current sources in the head, the effect of a single current source spreads to several electrodes. The result is a spatial low-passing of the original signal, leading to a “smearing” of the signal source and reduction in the spatial resolution. Thus most EEG setups for motor decoding only involve 64 or 128 electrodes. Setups with higher than 128 electrodes are uncommon.

EEG signal is traditionally separated into several frequency bands (delta: 0 – 4 Hz, theta: 4 – 7.5Hz, alpha: 8 – 13Hz, beta: 13 – 30Hz, gamma: 30 – 100Hz). Of particular importance to motor decoding is the brain oscillation in the alpha band over the motor and somatosensory cortex, also known as the *μ*-rhythm [16, 17]. It has been observed that there is a decrease of the signal power in the 8 – 13 Hz band when a subject is carrying out actual or even imagined movement [18, 19]. Similar observations can also be found in the lower beta band (12 – 22Hz). Although some components of the beta band oscillation may be harmonics of the alpha band signals, the common consensus now is that they are independent signal features due to having different topographic and timing characteristics [18, 20]. The mu-rhythm tends to focus on the bi-lateral sensorimotor area while the beta rhythm concentrates mainly on the vertex. Collectively, the modulation of the signal band power over the sensorimotor area is called sensorimotor rhythm (SMR).

**Fig. 2:**
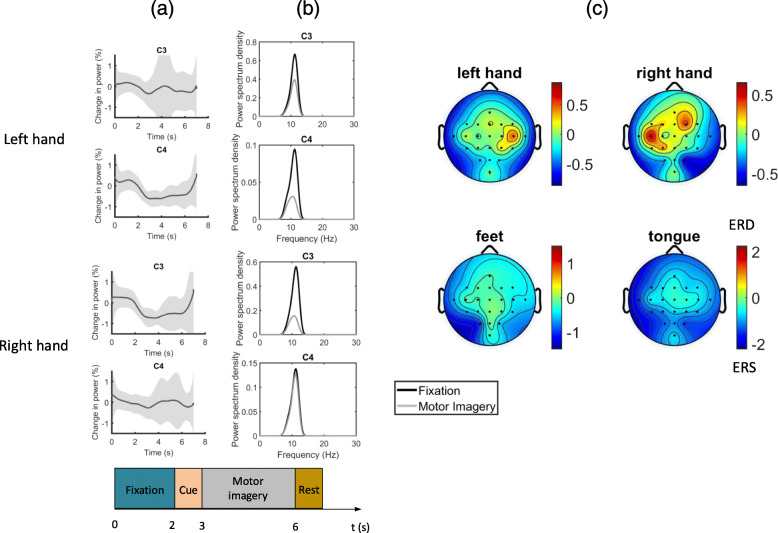
Examples of EEG features in motor decoding. EEG features from one of the subject from the BCI Competition IV 2a dataset [214]. **a** The time course of the change in band power of the EEG signal filtered between 8-12Hz, in left hand and right hand motor imagery, compared to a reference period (0-3s). The shaded regions show the standard deviation of the changes across different trials. The experimental paradigm is also shown below. **b** The frequency spectrum of the EEG signal during the fixation and motor imagery (**c**) the topography of the ERD/ERS distribution in different types of motor imagery

This decrease of band power coinciding with an event is called event-related desynchronization (ERD). The opposite is called event-related synchronization (ERS), which is the increase of band power coinciding with an event. ERD/ERS is typically calculated with respect to a reference period, usually when the subject is wakefully relaxed and not doing any task [21]:

$$ERD = \frac{R-A}{R} \times 100\% $$

where *R* is the band power during the reference period and *A* is that during the time period of interest. An example of ERD topography during motor imagery is shown in (Fig. 2).

The ERD topography during movement displays an evolving pattern over time [21]. ERD usually starts around 2 s before actual movement, concentrating on the contralateral sensorimotor area, then spreads to the ipsilateral side and becomes bilaterally symmetrical just before the start of movement. After the movement terminates, there is an increase of beta band power (i.e. ERS) around the contralateral sensorimotor area [21–23], also known as the “beta rebound”. The occurrence of beta rebound coincides with reduction in corticospinal excitability [24], suggesting the rebound may be related to the deactivation of the motor cortex after a movement terminates. Beta rebound occurs in actual as well as in imagined movements. An example of the beta rebound can be observed in (Fig 2a).

Different kinds of motor imagery (MI) produce different topograpies of ERD and hence are useful for decoding the motor intention of the subject. For example, imaging moving one’s hand will elicit ERD near the hand area of the motor cortex, which is in the more lateral position. On the other hand, imaging a foot movement will elicit ERD near the foot area in some of the subjects, which is closer to the sagittal line [25], as can be observed in (Fig. 2c). The beta rebound after MI also displays a similar somatotopic pattern [22]. Simultaneous ERD and ERS on different parts of the brain is also evident in some of the subjects. For example, some subjects showed ERD in the hand area and ERS in the foot area during a voluntary hand movement, and vice versa during a foot movement[22]. ERD may represent an activation of the cortical area controlling the motion while an ERS may represent an inhibition of other unintended movements. As we recall from the neurophysiology of motor control, the indirect pathway of basal ganglia contains mechanisms to suppress the thalamic activation to SMA to filter out unintended movements. There are characteristic patterns of ERD/ERS during different actual and imagined movements, thus by looking into those patterns we can detect and distinguish the motor intention of different body parts.

The most reactive frequency band at which ERD/ERS occurs may be specific for each subject and even for the type of motor imagery, and its topography may vary slightly across different EEG preparations. Therefore, signal processing and machine learning techniques are usually employed to adapt to the signal features of the subjects automatically.

One of the most important signal processing step in SMR-based motor decoding is the estimation of signal power in the frequency range of choice, typically in the alpha (8–12 Hz) and beta (12–30 Hz) band. There are many methods to achieve this. One of the simplest and most computational efficient method is band-pass filtering [3, 26]. The EEG signal is first band-pass filtered in the frequency band of interest, then the sum of the square of the signal is then taken as the power of the signal in the chosen frequency band. Sum-of-the-square is equivalent to the variance of the signal, so usually the variance of the signal is used instead. After taking the variance, a log-transform is commonly employed. The log-transform can serve two purposes. First, it transforms skewed data to make them more conforming to the normal distribution [27], which may help improve performance in some classification algorithms. Second, the log-transform emphasizes the relative change of the signal rather than the absolute difference (e.g. *log*(110)−*log*(100)=*log*(1100)−*log*(1000)), so it can perform an implicit normalization of the signal and improve the performance of the classifier.

One of the major drawbacks of the simple band-pass filtering approach is that it may be difficult to choose the best frequency band to perform the filter, as each patient has their own specific reactive band. To overcome this limitation, the adaptive auto-regressive (AAR) model is another commonly employed technique [28–31]. It models the signal at current time point as a linear combination of previous *p* points:

$$Y_{t} = a_{1,t} Y_{t-1} + a_{2,t} Y_{t-2} + \dots + a_{p,t} Y_{t-p} + X_{t} $$

where *Y*_*t*_ is the signal, *X*_*t*_ is the residue white noise and *a*_*p*,*t*_ the autoregressive coefficients. The core difference with the traditional AR model is that in the AAR model, the coefficients *a*_*p*,*t*_ are dependent on time and are calculated for each signal time point using recursive least square [32]. AAR coefficients from multiple electrodes are then concatenated together to form the feature vector used by a classification system. AAR coefficients can be seen as the impulse response of a system and so it contains information about the frequency spectrum of the modeled signal. Compared to the traditional band-pass filtering, spectrum estimation using AAR can be more robust against noise. One can also specify the number of spectrum peaks based on domain knowledge (each peak requires two coefficients). Another advantage is that there is no need to choose a subject-specific frequency band beforehand as all model coefficients are used for classification. Another way to choose the subject-specific frequency band automatically is to use a filter bank that consists of multiple band-pass filters in different frequencies. After filtering, the most informative frequency band and channels are then selected using some performance metrics, e.g. whether deleting those feature will lead to a reversal of the classification label [33, 34].

Due to the volume conduction problem in the human head, a single current source often appears to be “smeared” across several EEG electrodes. Spatial filtering is usually employed to improve the spatial resolution of the EEG signal. Popular spatial filters include the common average reference (CAR) and surface Laplacian [35]. These methods re-reference the signals by subtracting the voltage at each electrode from the average (as in CAR) or from its neighbors (as in surface Laplacian).

$$V_{j}^{CAR} = V_{j} - \frac{1}{N} \sum_{N}^{k=1} V_{k} $$

$$V_{j}^{LAP} = V_{j} - \frac{1}{n}\sum_{k \in S_{j}} V_{k} $$

where *V* is the signal voltage, *N* is the total number of electrodes, *n* the number of neighboring electrodes, and *S* is the set of neighboring electrodes in surface Laplacian (LAP).

These filters enhance the focal activity by acting like a high-pass spatial filter. There are also other more advanced spatial filters proposed. For example, the popular common spatial pattern (CSP) [36, 37] works by finding a projection of the electrode voltage such that the differences in variance between two classes are maximized. A further variation of the method is to add in frequency information by filtering the signal by a set of filter bands and then calculate the CSP for each, and finally select the most informative feature through a mutual information criterion [38].

The performance of EEG-based motor decoding has been improving steadily over the years. While earlier studies can only distinguish between discrete types of motor imagery [39], recent studies have already achieve 2D [40] and 3D control [41–43]. Some of the latest studies even demonstrate that it is possible to decode different movements in the same limb [44, 45] or even individual finger movements [46].

Besides being used to replace the lost functions, EEG-based motor decoding can also be used a tool for rehabilitation. For example, it can be used to control a robotic hand to assist in active hand training in post-stroke rehabilitation [4, 47, 48]. This application of motor decoding as a tool for training is a very promising area, as it can potentially extend its use to a wider population.

#### Electrocorticogram (ECoG)

ECoG is the measurement of the electrical signals from the brain on top of the dura, but underneath the skull. ECoG measurement is commonly performed before an epilepsy surgery to delineate the epileptogenic area and identify important cortical regions to avoid during a resection [49]. ECoG signal is not affected by the skull and thus tends to have a higher temporal and spatial resolution than EEG. It also has a larger bandwidth (0 to 500 Hz) [50, 51] and higher amplitude (maximum ∼500 *μ*V [52]). Therefore, generally ECoG has a higher signal-to-noise ratio than EEG although it is also more invasive.

ECoG and EEG likely arise from the same underlying neural mechanisms therefore they share many similarities with each other. Howevers, there are two major signal features in motor decoding that are unique to ECoG and are specifically exploited. The first is the change of signal band power in the high gamma band (≥75Hz). Many studies have suggested that the high gamma band contains more informative features for motor decoding compared to the alpha and beta band, which are typically used in EEG decoding [53–57]. Interestingly, the high gamma band tends to increase during movement, unlike the alpha and beta band, which typically show desynchronization (i.e. decrease in power). Therefore, high gamma power may be produced by a different neural mechanism than the one that produces the alpha and beta desynchronization.

Another unique feature is the low-frequency amplitude modulation of the raw ECoG signal, coined as the Local Motor Potential (LMP) by Schalk et al. [30, 51]. It was found that the envelop of the raw ECoG shows a striking correlation to the movement trajectory of the human hand, as measured by a joystick. The amplitude also shows a cosine or sine tuning in relation to the movement direction, similar to what have been observed in intra-cortical recordings. Since this discovery, many group have incorporated the LMP into ECoG motor decoding in addition to other high frequency features (e.g. [53, 56, 58, 59]). The LMP is a very low frequency component (2-3 Hz) of the raw ECoG signal. It is usually extracted by Guassian low-pass filter, running average [30, 53, 59], or the Savitzky-Golay filter [58, 60, 61].

Due to the robustness of the LMP signal, usually a simple linear regression is sufficient to decode the motor intention in many of the previous studies (e.g. [51, 62, 63]), although a feature selection or regulation step may be needed to first remove the uninformative features. A recent study using deep neural network also show promises [64], however its improvement compared to classical techniques is not always significant.

Because ECoG has a better resolution and higher signal-to-noise ratio, it tends to produce better and finer results than EEG in motor decoding. Beside decoding the movement of different body parts as in EEG [65, 66], different hand gestures can also be distinguished [56, 67]. Using the LMP in addition to frequency features, position and velocity of 2D arm movement can also be decoded from ECoG signals [30, 51, 58]. Subsequent studies even demonstrate that continuous finger positions can also be decoded [54, 59, 61, 63, 64, 68]. The correlation coefficient between the predicted and actual finger movement can reach from 0.4 to 0.7 in some of the recent studies [61, 64].

The large majority of studies in ECoG motor decoding are performed on epilepsy patients without a specific movement disorder or limb injury. However, one of the strongest motivation for motor decoding is that it can compensate the lost motor function of a patient. Given that the brain may re-organize due to disease or injury, it is vitally important that the decoding experiments be repeated on those patient population as well to see if similar decoding performance can be achieved. There are only a few studies to try ECoG motor decoding in stroke patients [57, 69] and paralyzed subjects [70], but the results are encouraging.

#### Intra-cortical recordings

Penetration into the cortical tissue offers the closest proximity to the neurons and produces the most precise signal. Since the discovery of the directional tuning property of the neurons in the motor cortex [71], a lot of studies have been trying to decode motor intention from intracortical recordings, first in non-human primate (NHP), then in human subjects in recent years. Our review will focus on intracortical decoding in human as it presents some unique challenges compared to NHP, and it is also where the technology will ultimately be applied.

Penetrating electrodes for motor decoding are usually implanted into the primary motor area of the brain. There is a structure in the precentral gyrus resembling a “knob” that houses a majority of the neurons responsible for motor hand function [72]. This “motor hand knob” is typically used as the target for electrode implantation (e.g. in [73–77]). Another potential target for implantation is the posterior parietal cortex (PPC). Although PPC has long been proposed to play an important role in the associative functions, in recent years more and more evidence suggests that it also encodes the high-level motor intention of the subject [78]. A recent study suggests that the goal and trajectory of the movement can be decoded from neural activities in human PPC [79].

**Fig. 3:**
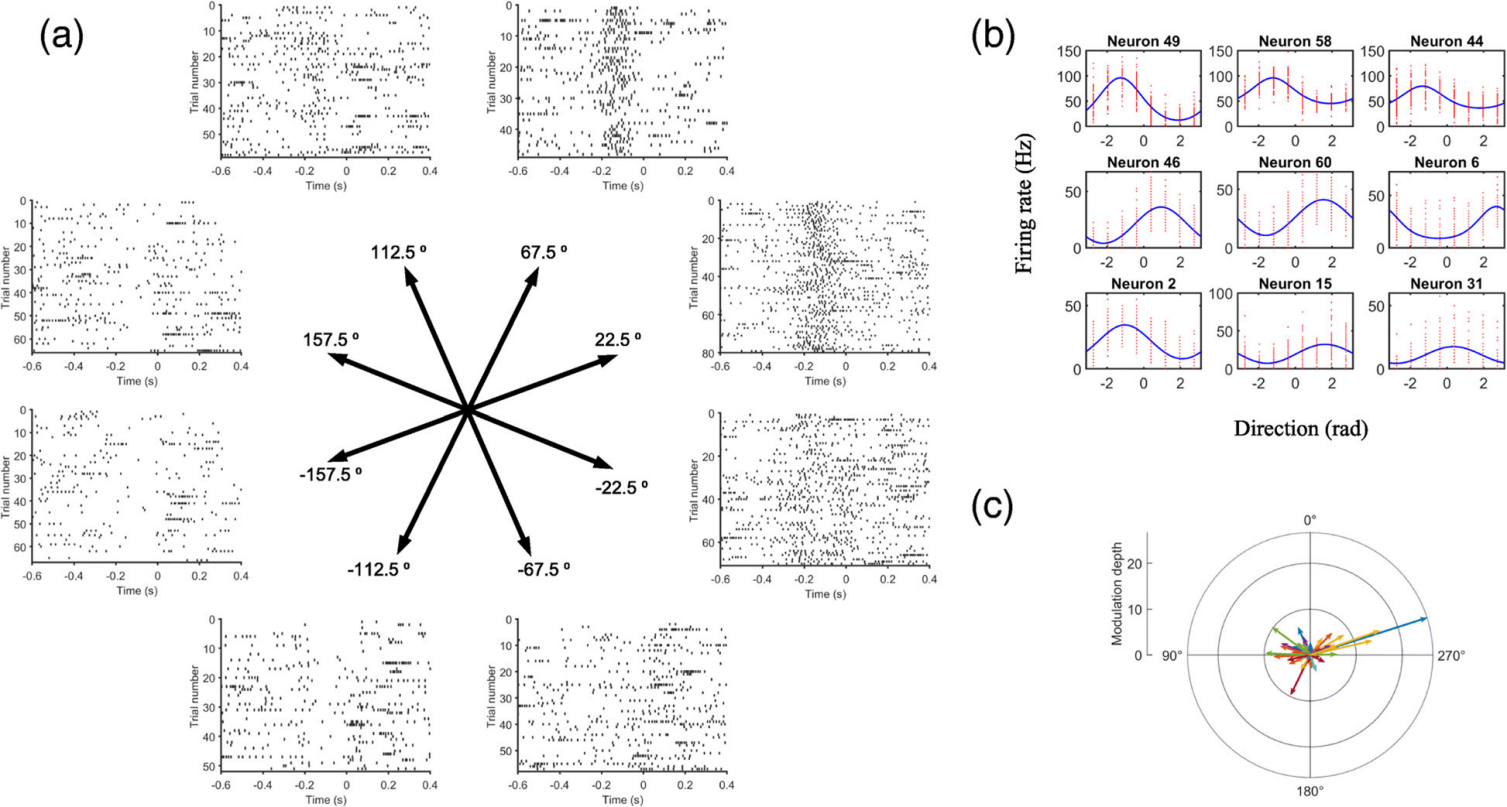
Examples of directional tuning in intra-cortical signals. Diagrams showing the directional tuning properties of the neurons in non-human primate M1 from the data in [215, 216]. **a** Spike raster plots from one of the neurons (Neuron 31). Each plot shows the spike timing of the neuron aligned to the time point (t=0) at which the movement speed of the hand exceeds a pre-defined threshold. Each dot in the plot represents an action potential. Different plots indicates the neuronal activity when the hand is moving in different directions. **b** The von Mises tuning curve of some of the representative neurons. **c** The preferred direction of all the neurons. The length of the vector represents the modulation depth of the neuron, here defined as the magnitude of the tuning curve divided by the angle between the maximum and minimum point on the curve

One important property exhibited by the neurons in the M1 is directional tuning. Some of the neurons are broadly tuned to a particular direction. They discharge the strongest when the movement is in their preferred direction, but they will also discharge less vigorously when the movement is in other directions. Their firing rates represent the length of their preferred direction vector. When the vectors of those neurons are summed together, it indicates the final direction of the movement. This population encoding of movement is a striking property of the nervous system. Similar analog of population encoding can also be found in the super colliculus representing the direction of eye movement [80]. An example showing the directional tuning property of M1 in a non-human primate is shown in (Fig. 3).

Currently, the only FDA-approved, commercially available microelectrode array for temporary (<30 days) intracortical recordings is the Neuroport System (Blackrock Microsystem, Inc, USA). As a result, majority of the work on human intracortical decoding are performed on that platform. Other intracortical electrodes do exist but they are either mainly for acute intraoperative monitoring (e.g. Spencer Depth Electrode, Ad-Tech; NeuroProbes, Alpha Omega Engineering Ltd; microTargeting electrodes, FHC), or for EEG applications (e.g. DIXI Medical Microdeep Depth Electrodes).

The activities of the neurons in the implanted site are represented by their action potentials, which manifest as spikes in extracellular recordings. Therefore, detecting the occurrence of a spike is often the first step in intracortical signal processing. There are many methods for spike detection [81, 82]. The signal is typically first band-passed filtered in the spike frequency band (e.g. 300-5000Hz), then various methods are used to transform the filtered signal to improve its signal-to-noise ratio (SNR). A detection threshold is then calculated to distinguish spikes from background noise. One of the most common spike detection methods is to use the root-mean-square of the signal

$$Thres = C* \sqrt{\frac{1}{N}\sum_{n=1}^{N}x[\!n]^{2}} $$

where *Thres* represents the detection threshold above which a signal time point is considered belonging to a spike. However, the RMS value may be easily contaminated by artifacts, so another way is to use the median to set the detection threshold [83]

$$ \sigma = median\left(\frac{|x|}{0.6745}\right) $$

$$ Thres = 4*\sigma $$

The non-linear energy operator is also another popular method [83]. It first transforms the signal such that the high frequency component is amplified to improve the SNR.

$$\psi(x[n]) = x[\!n]^{2}-x[\!n+1]x[\!n-1] $$

$$Thres = C\frac{1}{N}\sum_{n=1}^{N} \psi[x(n)] $$

Other more advanced techniques like continuous wavelet transform [84] and EC-PC spike detection [82] can offer a better accuracy but at a higher computational cost. Although there are a lot of ways to detect spike accurately offline, not every one of them are fast enough to be used in real-time. Therefore in online decoding the choices are usually limited to the simpler algorithms. Manually setting a threshold by an operator still remains one of the most commonly used method. Another popular method in online decoding is the RMS method due to its high efficiency.

An electrode may record signals from multiple neurons nearby. Isolating the activity of a single neuron (i.e. signal-unit activity) from this multi-unit activity usually leads to better results in motor decoding. This process is called spike sorting. There is a large body of literature on spike sorting that cannot be exhausted here. Interested readers are encouraged to consult other excellent reviews [85–87]. In practice, the most popular way to do online, real-time spike sorting is via template matching. A set of spike templates are collected during a period of initial recording, then subsequent spikes are classified by comparing their similarity with the templates. However, it may not be really necessary, or may even degrade the decoding result, to do online spike sorting. The spike clusters obtained from recordings may not be stable across different sessions of experiments. The total number of single units sorted from recording may change from sessions to sessions [79]. Thus a decoder trained on some sorted spikes may not work well on future sessions. Spike sorting may also introduce additional latency in online decoding, as accurate spike sorting is a computationally expensive process. In fact, many recent decoding studies do not use spike sorting at all, e.g. [79, 88–94].

A decoding algorithm reconstructs motor kinematics from neural activity. Since the discovery of the directional tuning property of motor neurons, one of the earliest decoding algorithm for intracortical spike signal is the population vector algorithm[95, 96]. In its simplest form, the firing rate of a neuron can be related to its preferred direction by

$$f = f_{0} +f_{max} cos(\theta-\theta_{p}) $$

where *f* is the neural firing rate, *f*_0_ and *f*_*max*_ are regression constants and *θ* and *θ*_*p*_ are the current and preferred direction respectively. However, for cosine function the width of the modulation is fixed. A more flexible tuning function that allows adjustable width of the modulation is the von Mises tuning function [97]:

$$f = b+k \; exp(\kappa cos(\theta-\mu)) $$

where *b*, *k*, *κ*,*μ* are the regression constants, and *θ* is the current movement direction. When *μ*=*θ*, the function will be at maximum, so *μ* can also be interpreted as the preferred direction of the neuron. Examples of the von Mises tuning curves are shown in (Fig. 3b).

The preferred directions of each of the neurons then can be summed together to predict the target direction [96].

$$P(M) = \sum^{N}_{i=1} w_{i}(M) C_{i} $$

where *C*_*i*_ is the preferred direction for the *i*-th neuron, and *w*_*i*_(*M*) is the weighting function combining the contributions of each neuron in direction *M* to the final population vector. However, this method requires a large number of neurons to be accurate and may lead to error if the distribution of the preferred direction is not uniform [98]. For example in (Fig. 3c), we can see that the preferred directions are not distributed evenly. For this reason, a simple linear regression scheme is usually employed instead in recent studies [73],

$$\mathbf{u} = \mathbf{Rf} = \mathbf{R} (\mathbf{R}^{T} \mathbf{R})^{-1}\mathbf{R}^{T} \mathbf{k} $$

where **R** is the neural response matrix (e.g. firing rate), **f** is the linear filter (or the regression constants) and **k** is the motor kinematic values (e.g. joint angles or cursor positions). It has been suggested that this regression scheme can provide more accurate prediction compared to the summation of preferred direction vectors, especially when those vectors are not uniformly distributed [98].

In recent years, the Kalman filter is usually employed instead of the simple linear regression (e.g. in [75–77, 99, 100]). The Kalman filter incorporates the information both from an internal process model and actual measurement to estimate the states of a system [101]. A Kalman gain variable is used to determine the “mixing weight” of the model and measurements. If the model is more accurate, then it will trust the model more. The same goes for the measurement. Kalman filter is especially useful if the states are not directly observable or if the measurement is very noisy, which are often both true in motor decoding. In motor decoding, the subjects usually lost their limb or ability to move, therefore the internal state (e.g. motor intention) of the system is not directly observable. The observable variables (e.g. neural activity) are also very noisy. A typical Kalman filter for motor decoding assumes no control variable and the system can be formulated as two linear equation [102, 103]):

$$\begin{array}{@{}rcl@{}} \vec{x}_{t} &=&A\vec{x}_{t-1} + \vec{w}_{t-1}  \\ \vec{y}_{t} &=& C\vec{x}_{t} + \vec{v}_{t}  \end{array} $$

where *x* is the state of the system one want to decode, e.g. joint kinematics or cursor position. *y* is the observed variables, e.g. neural firing rate. $\vec {w}_{t}$ and $\vec {v}_{t}$ are the process and measurement noises drawn from *w*_*t*_∼*N*(0,*Q*) and *v*_*t*_∼*N*(0,*R*) respectively. *A*, *C*, *Q* and *R* are the Kalman constants that need to be defined according to the decoding model. For the internal state *x*, if it is a cursor position, it can be expressed as

$$x_{t} = [pos_{t}, vel_{t}, 1]^{T} $$

With the model defined, the Kalman gain *K* and the estimation error covariance *P* then can be updated with the typical two-step update equations:

Predict:

$$\begin{array}{@{}rcl@{}} \hat{x}^{-}_{t} &=& A \hat{x}_{t-1} + B u_{t} \\ P^{-}_{t} &=& A P_{t-1} A^{T} +Q  \end{array} $$

Update:

$$\begin{array}{@{}rcl@{}} K_{t} &=& \frac{P^{-}_{t}C^{T}}{CP_{t}^{-}C^{T}+R} \\ \hat{x}_{t} &=& \hat{x}^{-}_{t} + K_{t}(y_{t}-C\hat{x}_{t}^{-}) \\ P_{t} &=& (I-K_{t}C)P_{t}^{-}  \end{array} $$

where $\hat {x}^{-}$ and $\hat {x}$ are the *a prior* and *a posterior* state estimates respectively. *u* is the control variable. Typically it is set to 0 in motor decoding, here we have included it for completeness.

One crucial aspect of performing online motor decoding is the training and re-calibration of the decoding model. Although the neural features for similar movements are relatively stable within a few days [104], the neural tuning curve may start to change when the subject is learning to perform a new task [105]. It is also very difficult to track the same neuron for an extended period of time [106, 107], due to the micro-movement of electrodes and fluctuations of other noise sources. Furthermore, training data are often acquired in an open-loop fashion, meaning that no feedback is provided by the decoder during training. However, in actual decoding session, feedback is provided and the subject may attempt to change his motor imagery in order to “learn” the decoder. This may lead to changes in the underlying neural features [108]. Therefore, re-calibration of the trained model is often necessary and will be ideal if it can be performed online. A successful re-calibration method is the ReFIT-KF algorithm proposed by Gilja *et al* [109]. ReFIT-KF assumes the subject’s true intention is to move towards the target, so it can generate a pseudo-ground truth from the decoded result automatically even though the prediction of the current model may be wrong. It can then calibrate the model using the estimate ground truth to adapt for the instability of the neural signals. It is able to produce better results than Kalman filter alone [92,93,109].

Due to the more robust signals obtained by intracortical recordings, it has been utilized successfully to help tetraplegia patient control the environments in various ways, including 2D cursor control [73,76,94], virtual and real prosthetic hands [77,79,92,110,111] and functional electrical stimulation of the patients’ own paralyzed hands [90,91,93].

### Peripheral decoding of limb movements

Signals from the central nervous system (CNS) eventually arrive at the peripheral nervous system (PNS) and drive the contraction of different muscle fibers. Compared to CNS, signals in the peripheral structures are usually more specific. They contain detailed instructions on the contractions of individual muscle fibers, therefore potentially can enable dexterous prosthetic control. Surgeries involved in peripheral interface are usually less complicated than those involving the intracortical structures. Therefore, many studies are also devoted to motor decoding in the peripheral structures.

#### Peripheral nerve recordings

Peripheral nerves contain the low-level neural signals sent to activate the contraction of specific muscles. Previous studies on peripheral neural recording mainly focus on afferent sensory information because it is not easy to get efferent signals in anesthetized animals [112]. However, in recent years, more studies have appeared trying to explore the possibility of decoding efferent peripheral nerve signals for prosthetic control. Because the peripheral nerves contain low-level information targeting each muscle, it may be possible to regain high-dexterity and naturalistic control by exploiting this rich information.

One of the major challenges in peripheral nerve recordings is accessing the axons in the nerves. Axons in spinal nerves are bundled in fascicules and multiple fascicules are grouped together to form a peripheral nerve. Those axons are enclosed in three sheaths of connective tissues – the epineurium that covers the entire nerve, and the perineurium that encloses a fascicle and the endoneurium that holds the neurons and blood vessels together within a fascicle. Due to these multiple layers of lamination around an axon, the amplitude of a peripheral nerve signal is usually very small, can be around 5 – 20 *μ*V [112].

There are multiple electrode configurations designed to get a better signal from the peripheral nerves [113]. The cuff electrode [114], as its name suggests, works like a cuff to wrap around a nerve. Its main advantage is that it causes minimal damage to the neural tissues as it does not require any incision on the nerve itself. However, since it only measures the electrical potential at the surface of a nerve, it can only obtain a grand summation of the neural activity in different fascicles. Another variation of the cuff electrode is the flat interface nerve electrode (FINE) [115]. It works like a clip to apply pressure on the nerve and make it flattened into an oval shape, thus increasing its surface area and reducing the distance from the electrode to the fascicles. There are also other types of electrodes that are implanted into the nerves. They offer higher selectivity due to their direct contact with the fascicles. However, they are also more invasive and may cause more damage to the nerve. The longitudinal intrafascicular electrodes (LIFE) are long, thin wires implanted longitudinally into the nerve fascicles [116]. On the other hand, the transverse intrafascicular multichannel electrodes (TIME) are implanted transversely into the nerves, accessing multiple fascicles at the same time. There is also the Utah Slanted Electrode Array [117], which consists of an array of electrodes with different lengths, such that when the array is inserted into the nerve, the tip of the electrode can get into contact with different fascicles. Recently, there is also development of the regenerative peripheral neural interface (RPNI) [118], which uses a muscle graft to wrap around severed fascicles endings. The nerve endings grow into and innervate with the graft, creating a new interface for acquiring neural signal. Of the different types of electrodes introduced, only the cuff electrode is currently used in commercial FDA-approved systems for vagus nerve stimulation (e.g. VNS Therapy, Cyberonics, USA). Most of the others are still in research or undergoing clinical trials [119].

Studies on the human decoding of peripheral signals are still very limited, partly due to the challenge of acquiring nerve signals with sufficient SNR, and may also due to the cross-talk between neural signals and EMG, as the peripheral nerves are usually located in close proximity with the limb musculature. The majority of existing studies focus on upper limb decoding, as upper-limb amputation tends to have a bigger impact on the everyday life of the patients. Neural recording are performed on the ulnar, medial and/or the radial nerve. Different types of electrodes are used, but the more common ones in human decoding are the Utah slate electrode (e.g. in [120,121]) and the LIFE (e.g. [122*–*124]).

The analysis of peripheral signals commonly involves the detection of action potentials in the nerve. The detection procedures are similar to those used in intracortical studies, but the step of clustering spikes is not usually performed. Due to the low SNR of the peripheral signals, sometimes they need to be first de-noised (e.g. by wavelet [124]) before detection. The firing rate of the action potential can then be fed into a regressor (e.g. in [103,120*–*122]) or a classifier (e.g. in [123,124]) for decoding. The difference in using a regressor or a classifier lies in whether a discrete gesture or a continuous joint trajectory is decoded.

Support-vector machine (SVM) is the most commonly used classifier for peripheral decoding (e.g. in [123,124]). For regressor, simple linear regression or a Kalman filter have been used ([103,120*–*122]). Kalman filter allows the online recursive update of the model in real-time, and is especially helpful when the measurement of the target variable is noisy (as often in the case of motor decoding, since it is not possible to measure the actual movement of the missing limb).

The issue of obtaining ground truth for training the decoder is also very important. While for discrete grasp type classification, it may be sufficient to ask the subject to imagine holding a particular grasp, for position decoding a more precise approach have to be used. One common solution is to show a shadow hand on a screen, and ask the subject to try to follow the movement of the hand, either through a manipulandum controlled by the mirrored movement in the intact hand [121] or through imagined phantom limb movements only.

Currently, the performance of human peripheral nerve decoding is still not very satisfactory, partly due to the difficulty in obtaining clear signal and EMG cross-talk. In discrete grasp classification, a 4-class classification task with 3 grasps (power grip, pinch grip, flexion of little finger) and rest have obtained 85% accuracy [124], but state-of-the-art surface electromyogram (EMG) can already distinguish between 7 gestures [125]. Regression-based decoding enables proportional control of a prosthetic hand, and hence can be more intuitive. Decoding based on Kalman filter is able to classify 13 different movements offline, but only 2 movements can be decoded online successfully due to the cross-talk between different degree-of-freedoms (DoFs) [121].

The peripheral nerves offer a promising target for motor decoding. It is more downstream in the motor control pathway and contains more specific information about muscle activities. This property can be potentially exploited to enable high dexterity control. Access to peripheral nerves is also relatively easier than intracortical structures. However, peripheral recordings are plagued by their low SNRs due to the multiple levels of lamination around an axon. This may be improved by better electrode designs, and ultra-low-noise neural amplifiers that can resolve the small amplitude of the nerve signals (e.g. [126]).

#### Electromyogram (EMG)

**Fig. 4:**
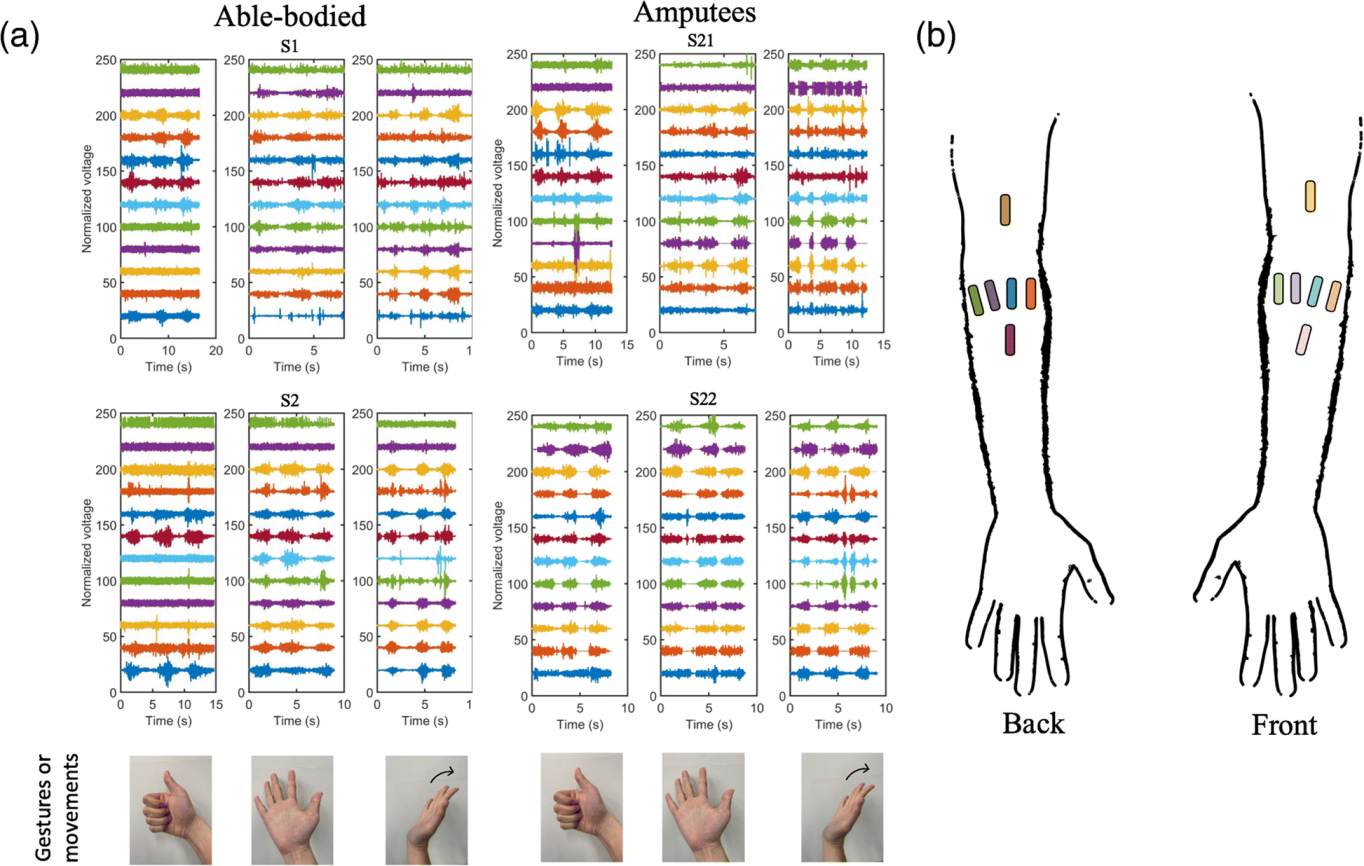
Examples of EMG signal in different hand gestures. Diagram showing EMG signals from 12 surface electrodes in 3 different hand gestures. The original data are from [217]. **a** EMG signals from both able-bodied and amputee subjects. The last row shows the hand gestures performed for their respective EMG segments. **b** Locations of the 12 EMG electrodes

EMG signals are the sum of the electrical activities of the muscle fibers, which are triggered by spike trains, i.e. impulses of activation of the innervating motor neurons. EMG signals can be measured in two ways, either on the surface of the skin above a muscle (surface EMG), or directly inside a muscle fiber using a needle electrode (intramuscular EMG). An example of EMG data in different hand gestures is shown in (Fig. 4).

Myoelectric signals have been used as the control source for decades in prostheses, in which muscle signals are recorded and translated into control commands to induce prosthesis motions. Intramuscular EMG signals are believed to be of a higher resolution and less susceptible to cross-talks compared with surface EMG because of its more invasive electrode deployment and direct targeting of specific muscles.

Despite decades of research and development, amputees still do not use state-of-the-art myoelectric prostheses more frequently than the basic, body-powered hooks [127], and an estimate of 40% of upper-limb amputees actually reject using a prosthesis [128]. One primary limitation of clinically available hand-prosthesis is the number of simultaneously and proportionally controllable degrees of freedom (DoFs), which is rarely greater than 2 [129,130] and has focused mostly on wrist DoFs without the hand [131], although functions of hand-movement are more essential for daily living.

Myoelectric control can be categorized into direct control and pattern recognition control. Direct control refers to the type of methods that use the amplitude of two surface EMG inputs from an antagonistic muscle pair to control the two directions (ON and OFF) at a prosthetic DoF. Due to the inadequate remaining musculature, signal crosstalk contamination, and attenuation of deep muscle signals at the skin level, the number of independent myosites in the residual forearm is typically limited to two, only allowing the control of one DoF at a time. As a result of this constraint, patients need to toggle between modes using quick co-contraction at the myosites to sequentially control multiple DoFs. Pattern recognition control relies on machine learning algorithms to train a separate classifier for each DoF. Multiple classifiers have been proposed and evaluated, including quadratic discriminant analysis [132], support vector machine [133], artificial neural network [134], hidden Markov models [135], Gaussian mixture models [136], and more. However, as training of the computational models involves the movement of only 1-DoF, the trained classifiers do not support simultaneous control of multiple DoFs. A more promising approach based on machine learning is adopting a regression-based control scheme (instead of classification) that inherently facilitates continuous control (as opposed to ON and OFF), in which a linear or nonlinear mapping from EMG signal features to the changes of prosthesis DoFs is learned. Commonly used methods for this purpose include artificial neural networks [137], support vector machine [138], and kernel ridge regression [131]. A major shortcoming of regression-based control is the requirement for large amount of training data that include an exhaustive combination of movements of all prosthesis DoFs, which is impractical to be clinically implemented.

One of the fundamental issues with EMG based prosthesis control is the scarcity of independent signals with which to control prosthesis DoFs. EMG signals are inherently heavily correlated and lacks the resolution and the information capacity needed for simultaneous and proportional control of multiple DoFs. A potential solution to this problem is to record motor commands directly from the peripheral nerves, such as ulnar and median nerves that directly innervate all five fingers. However, this comes at the costs of invasive surgical implantation of electrodes and the risks of tissue infection and nerve damage.

There have been works to extract more invariant and independent information from EMG signals without invasive recordings. One major group of the efforts focuses on extracting muscle synergy features from EMG recordings, i.e., the complex muscle activation patterns that are executed by users as high-level control inputs regardless of any neurological origin [139]. Muscle synergies are believed to be capable of describing complex force and motion patterns in reduced dimensions and can be used as a robust representation for decoding outputs consistent with user’s intent. Non-negative matrix factorization (NMF) [140] has been commonly used to extract muscle synergies from multichannel EMG signals for simultaneous and proportional control of multiple DOFs [137,141*–*143]. Another group of works focuses on directly extracting the neural codes of motor neuron activities that govern the muscle movements through the nerve pathway. This normally requires advanced recording setups such as high-density EMG with a sufficient number of recording sites that are closely spaced. A number of algorithms have been proposed to extract the underlying neural information [144,145]. Among them, convolution kernel compensation (CKC) has been most extensively used as a type of multichannel blind source separation method [146*–*149]. Despite the promise of extracting neural contents from high-density EMG signals, the demonstration of utilizing such scheme in online experiments remains difficult. More in-depth investigation and significant efforts are needed to build neural interface and achieve direct neural-based control based on this framework.

### Decoding of speech motor activities

Although this review mainly focuses on the decoding of movement in the extremities, recently there are also another line of research in decoding motor speech activities [150,151]. Speech production is a complex process involving multiple areas of the brain and dozens of muscles fibers. The muscle activities need to be highly coordinated to produce different speech sounds (i.e. phonemes) which concatenate together to form intelligible words and sentences.

Multiple brain regions are associated with language production [152], but there are two major areas that have received more attentions in speech decoding. The left ventral premotor cortex has been suggested to represent high-level phonemes in speech [153,154], while the ventral sensorimotor cortex contains rich representations of different speech articulators (e.g. lip, tongue, larynx etc.) [155,156]. Therefore most of the decoding efforts concentrate on these two brain regions.

Historically, various neural signals have been exploited to decode speech. EEG is non-invasive but its low signal-to-noise ratio and EMG contamination from facial muscles make it very difficult to be used for decoding speech [151]. There has been some success in using multielectrode array to decode phenomes from multi-unit activities [157]. However, the cortical representation of speech articulators cover a large area that may not be suitable for the very localized recording region of a multielectrode array [156,158]. Furthermore, speech decoding often require overt speech to serve as the ground truth, and that requires the subjects to be capable of speaking clearly. It is difficult to justify implanting penetrating electrodes in the otherwise healthy eloquent cortex to conduct experiments. Currently, ECoG obtains a greater success in speech decoding due to its high signal quality and less invasive nature. ECoG recordings are also commonly employed during brain resection to avoid damage to the eloquent cortex, so it is well-integrated into existing surgical procedures. Studies using ECoG for speech decoding mainly focus on the high gamma band (70-170Hz), as it has been shown that the high gamma activity correlates strongly with ensemble firing rate [159].

Earlier speech decoding efforts have focused on the direct decoding of simple words or phonemes [150*,*^157^*,*^158^*,*^160^*–*162], but their performance is not very satisfactory. Decoding from a limited dictionary or phoneme set may produce a higher accuracy (e.g. >80% for 10 words [160] or 9 phonemes [157]), but it can only cover a very narrow range of human spoken expressions. Studies trying to decode the full range of English phonemes result in a lower classification accuracy (10-50% [150,155*,*^162^]). The low classification accuracy can be partly mitigated by incorporating a pronunciation dictionary and language model (e.g. in [150]), which can limit the output of the decoder to more probable words.

On the other hand, recently attentions have been shifted to focus more on decoding the intermediate representation of speech (e.g. articulator movements) rather than decoding phonemes directly. Part of the shift may be motivated by the growing body of evidence suggesting that the speech motor cortex is able to generate differential activation patterns encoding the kinematics of speech articulators [156,163*–*165]. Advances in deep learning has made the prediction of articulator trajectories from acoustic signal (i.e. acoustic-articulatory inversion) accurate enough to act as the ground-truth for decoding, as the traditional ways of implanting coils or magnets in the mouth via articulography is invasive and not compatible with neural recordings [166]. In one very recent study [167], a deep neural network is used to decode ECoG features to articulator trajectories. The trajectories are then decoded by another neural network to acoustic features (e.g. pitch, mel-frequency cepstral coefficients etc.), which are then converted to audible voice using a voice synthesizer. Even mimed speech can be decoded, although with a lower accuracy. In another study [168], ECoG features are decoded into mel-scaled spectrograms directly using a neural network, then a neural network vocoder is used to construct the spectrogram into audible waveforms. These recent results show great promises in decoding human speech from ECoG signals. A summary of the different methods of motor decoding is shown in Table 1.

**Table 1:** Comparison of different methods for motor decoding

	Cortical			Peripheral	
	EEG	ECoG	Intra-cortical	Peripheral nerves	EMG
Decoding site	Scalp	On the surface of the brain	Penetrated into cortical tissues (e.g. PPC, M1)	Peripheral nerves (e.g. ulna, median, radial nerves)	Muscles
Types of electrode	Disk electrodes	Flexible electrode array	Utah array	Cuff, intra-neural electrodes	Surface electrodes,needle electrodes
Typical spatial resolution [14,169*–*173]	5-9 cm	<5 mm	3-5 *μ*m	0.5-2 mm	>10 mm
Frequency spectrum	0.5-100Hz	0-500Hz	100Hz-20kHz	0.1-10kHz	0.1Hz-10kHz
Decodable intention	Movement of different body parts, 2D and 3D direction of movement, different movements of the same limb, individual finger movement	Movement of different body parts, different hand gestures, 2D position and velocity of movement, continous finger position	2D direction of movement, different hand gestures	Different hand gestures	Different hand gestures, proportional control of grasps
Signal-to-noise ratio	Low	Medium	High	Low	High
Signal feature	Bandpower, ERS/ERD	Bandpower, LMP	Spike firing rate, LFP	Action potential firing rate	Various signal features (e.g. RMS, variance, mean absolute value etc.)
Invasiveness	Low	High	Very high	Medium	Low
Advantages	Non-invasive, easily deployable	Fine-grained and robust feature, mature surgical procedures as part of epilepsy treatment	Fine-grained and robust feature	Less invasive, potentially contains detailed information about muscle activations	Non-invasive, mature technology, easily deployable
Disadvantages	Low signal-to-noise ratio, high variability of features between sessions, time-consuming to setup	Invasive, long-term implantation not common	Very invasive, require implantation surgery	Low signal-to-noise ratio	Limited DoF, exessive cross-talk between different channels

### Challenges and future direction

Although great strides have been made in decoding human motor intention, there are still some significant challenges remain to be solved. One of the biggest challenge preventing the adoption of motor decoding outside the laboratory is the limited longevity of the decoding model. Typically, some calibration session is needed to collect data to train the decoding model, then the model is tested on subsequent sessions on the same or next few days. While it is acceptable in a scientific study due to the limited time and clinical resources available, in actual daily use, the trained model must be able to maintain its performance for an extended period of time.

The limited longevity can be due to several reasons. First is the instability of the electrode interfaces. Micro-movement of the electrodes may cause a shift in the feature space. If the decoder is not robust enough, this shift may result in a deterioration of the decoding performance. Another reason is the different environment noises injected into the acquired signals. Neural signals used for decoding usually have a very small amplitude and thus are susceptible to interference by environment noises. A cell-phone, fluorescence lamp or other electrical appliances all inject various types of noise in the acquired signal. As the subjects are performing various tasks in daily lives, they may come into the influence of different noise sources not covered in the trained data set and results in performance degradation. The third reason is the slow build up of immune response on the electrode interface. Glial scars may encapsulate the electrode and increase its impedance [174]. Neurodegeneration as a result of immune response will also lead to a weaker signal [175]. The model longevity problem is multifaceted and must be carefully addressed. First, a better electrode design can help secure the electrode onto its anchoring structure and reduce their relative movement. An implantable solution will also produce more stable feature than one that requires repeated dismantling and reinstallation every time (e.g. EEG and EMG). Second, the model should be trained with more robust features and tested in an environment typical of its everyday use. A shielded chamber may help acquire very clean signals that are good for the demonstration of a prototype. However, it is unlikely that the same quality of signals can be acquired in everyday environment. Thus it is also important to consider how a decoder is tested rather than just looking at offline numerical metrics. Thirdly, advancement in the electrode materials or special organic coatings can potentially reduce its immune response [176]. A flexible instead of rigid electrode may also cause less neuronal damage and inflammation [177,178].

The second challenge is how to account for the difference in features during open-loop training and close-loop control. The training dataset is typically obtained in an open-loop fashion, meaning that the subjects are instructed to carry out a particular motor imagery without any feedback. However, in actual use the system will provide feedback to the subject based on the decoder outputs. When the decoder output is wrong, the subject may try to correct it deliberately, and that may lead to discrepancy in the offline and online performance [179]. One of the solutions is to introduce a small calibration session with feedback at the beginning of the testing session, like in many EEG-based motor decoding studies. The original model is trained with an open-loop paradigm, then the model is further fine-tuned with feedback in the calibration session. However, this is only possible if a clear ground truth is available. For the case in which the ground truth is not available, e.g. in the case of a tetraplegic patient where it is very difficult to know the true intention of the subject, the ReFIT algorithm is another approach to address this problem [109]. The basic idea of the ReFIT algorithm is that it tries to construct a pseudo ground truth by assuming that the subject is constantly trying to correct the wrong output of the decoder. Thus the directional vector of the motor intention is taken to be always pointing towards the target from the current cursor position. Using this method, it is possible to train a decoder from scratch with as few as 3 min of data [94]. Online calibration with feedback can offer a more realistic prediction on how the decoder is able to perform in real-life. This approach can also let the decoder quickly adapt to any shift in the feature space due to change in the electrode interface or environmental noises. However, online calibration demands that the model can be updated quickly, which puts an constraint on the complexity of the decoding model. More research is needed to study how to update the decoder efficiently in real-time.

Besides advancement in decoding algorithms, development of new electrodes and neural amplifiers also play a very important part in advancing motor decoding. Recent trends in electrode development mainly focus on improving four areas of electrode design: density, flexibility, biocompatibility and connectivity. Denser electrode can improve the spatial resolution of neural recordings. High-density electrode has been created from silicon wafer and carbon fiber monofilament [180,181]. Electrode material with a flexibility closer to that of brain tissues can reduce neural damage and inflammatory response. Many flexible polymers have been used to make neural electrode, including polyimide [182,183], parylene [184], PDMS [185] etc. Biocompatibility is always an important issue in electrode design because inflammatory response and encapsulation deteriorate signal quality over time and undermine the quality of chronic neural recordings. Strategies to improve biocompatibility including using inert metals like gold or platinum, using flexible materials to reduce tissue damage, or coating the electrode with biocompatible materials like conducting polymer [186] and carbon nanotubes [187]. Read-out connection from the electrodes will also quickly become a problem when the density and number of electrode continue to increase. Incorporating transistors into the electrodes directly to enable connection multiplexing is one of the ways to mitigate this problem [188,189]. Readers interested in neural electrode designs are suggested to consult other more in-depth reviews in this area [119,172,176,177,190].

Development of neural amplifiers also plays a very important role in advancing the science of motor decoding, as we first need to acquire a clear neural signal before any processing and decoding can be done. There are multiple lines of research trying to improve the different aspects of the amplifier design. Firstly, the power consumption of an amplifier can be reduced by resource sharing (e.g. one amplifier sharing multiple electrodes [191] or multiple amplifiers sharing one analog-to-digital convertor [192]), power scheduling (e.g. switching off unused components [193], dynamically adjusting the amplifier parameters [194]), or reduction of supply voltage [195]. Secondly, the channel count can be increased by multiplexing or integrating amplifiers directly with the electrodes [191,196]. Thirdly, the circuit noise can be reduced by trimming [197], chopping [198,199], auto-zeroing [200] or frequency-shaping [201] etc.. Fourthly, wireless transmission of power or data can be achieved by an inductive link [193,202,203], short-distance power harvest [193,204] or even ultrasound [205]. Finally, the functionality of the amplifier can also be expanded by integrating more signal processing on-chip, e.g. spike detection [203], spike sorting [206,207] and data compression [208,209]. Interested readers are encouraged to consult other more focused reviews in this area [210*–*213].

## Conclusions

Every year, a large number of patients suffer from various degrees of movement disability due to amputation or neurological disorders. Their everyday lives and works are severely affected. With modern neurotechnology, it is now possible to intercept and decode the motor intention at different points along the neuro-muscular control pathway and use that information to drive a prosthetic device to restore movement. In this paper, we have reviewed the various signal features and techniques to decode motor intention in human. Although motor decoding performance is improving steadily with the advancements in electrode configurations, neural amplifier designs and decoding algorithms, we are still very far away from the goal of achieving naturalistic and dexterous control like our native limbs. The eventual successful clinical application of motor decoding will depend on the concerted efforts of both healthcare and engineering professionals, and likely also needs to be tailored-made according to the conditions and ability of each patient. We hope our review can provide a useful overview of the current state-of-the-art in motor decoding, so that researchers interested in the field can be aware of the neural features that they can exploit, potential problems they may encounter and the available solutions that they can adopt.

## Data Availability

All data analysed during this study are included in their corresponding original articles [214*–*217].

## Abbreviations

AAR: adaptive auto-regression
CAR: common average reference
CKC: convolution kernel compensation CSP: common spatial pattern
CNS: central nervous system
DoF: degree of freedom
ECoG: electrocorticogram
EEG: electroencephalography
EMG: electromyogram
ERD: event-related desynchronization
ERS: event-related synchronization
FINE: flat interface nerve electrode
GPi: internal globus pallidus
LIFE: logitudinal intrafascicular electrodes
LMP: local motor potential
M1: primary motor cortex
NMF: non-negative matrix factorization
NHP: non-human primate
PMA: premotor area
PNS: peripheral nervous system
PPC: posterior parietal cortex
RPNI: regenerative peripheral neural interface
SMA: supplementary motor area
SMR: sensorimotor rhythm
SNR: signal-to-noise ratio
STN: subthalamus nucleus
TIME: transverse intrafascicular multichannel electrodes
VLo: oral part of ventral lateral nucleus
